# An association between TRP64ARG polymorphism of the B3 adrenoreceptor gene and some metabolic disturbances

**DOI:** 10.1186/1475-2840-10-89

**Published:** 2011-10-12

**Authors:** Aibek E Mirrakhimov, Alina S Kerimkulova, Olga S Lunegova, Cholpon B Moldokeeva, Yulia V Zalesskaya, Samai S Abilova, Nurmira A Sovhozova, Almaz A Aldashev, Erkin M Mirrakhimov

**Affiliations:** 1 National Centre of Cardiology and Internal medicine named by M. Mirrakhimov, T.Moldo 3, Bishkek, 720040, Kyrgyzstan; 2 Kyrgyz State Medical Academy named by I.K. Akhunbaev, Akhunbaev street 92, Bishkek, 720020, Kyrgyzstan; 3 Kyrgyz-Russian Slavic University named by B.N. Eltsyn; Kievskaya 44, Bishkek, 720000, Kyrgyzstan; 4 Institute of Molecular Biology and Medicine, T.Moldo 3, Bishkek, 720040, Kyrgyzstan

**Keywords:** metabolic syndrome, B adrenoreceptors, Trp64Arg polymorphism, abdominal obesity, dyslipidemia

## Abstract

**Backgrounds:**

B3 adrenoreceptors (ADRB3) are abundant in adipose tissue and play the role in its metabolism and lipolysis. Some variants of the ADRB3 gene may predispose subjects for the development obesity and metabolic abnormalities in the setting of modern sedentary lifestyle. ADRB3 gene polymorphism association with metabolic disturbances has never been studied before in the ethnic Kyrgyz population.

**Aim:**

To study an association between Trp64Arg polymorphism of the ADRB3 and metabolic syndrome (MS) components in an ethnic Kyrgyz group.

**Materials and methods:**

213 Ethnic Kyrgyz volunteers over the age of 30 were enrolled in the study. The assessment plan for each individual comprised of general physical and anthropometric exams as well as laboratory tests (glucose, lipid panel, insulin) and genotyping by Trp64Arg polymorphism of the ADRB3. MS diagnosis was consistent with modified ATP III criteria (2005). Logistic regression analysis was performed to test the potential independent association between Arg64 allele with obesity, abdominal obesity (AO) and arterial hypertension (AH).

**Results:**

Trp64Arg polymorphism of the ADRB3 was assessed in 213 individuals (145 men, 68 women) aged 30-73 (mean age 50.7 ± 7.6). Arg64 allele frequency was 0.239; ADRB3 genotype distribution among participants was: Trp64 homozygotes 54.5%, Trp64Arg 43.2% and Arg64 homozygotes 2.3%. There was an association between Trp64Arg и Arg64Arg genotypes and higher BMI, WC and obesity frequency (p < 0.00009), AO (p < 0.01), type 2 diabetes mellitus (DM) (p < 0.005) and lower high density cholesterol (HDL-C) level (p < 0.03). The logistic regression analysis showed the correlation of the Arg64 allele with obesity (OR 3.159; 95% CI 1.789-5.577) and AO (OR 1.973; 95% CI 1.118-3.481). The association between Arg64 allele and AH lost its significance after adjustment for obesity.

**Conclusion:**

Arg64 allele of the ADRB3 gene in the studied group has an association with MS components such as obesity, AO and decreased HDL-C level.

## Introduction

Changes in lifestyle of the world's population, which include increased intake of high-calorie food together with low physical activity, promote the rising prevalence of metabolic syndrome (MS), consisting of such risk factors as glucose metabolism abnormalities, dyslipidemia, arterial hypertension (AH) and obesity [1]. The presence of MS substantially increases the risk of the subsequent development of cardiovascular diseases [2] and type 2 diabetes mellitus [3] (DM).

Genetic predisposition plays a crucial role in the development of obesity besides traditionally acknowledged risk factors such as overeating and a sedentary lifestyle. Attempts to identify genes responsible for obesity were focused mostly on white and brown adipose tissues [4], which play an essential role in the regulation, storing and energy expenditure in mammals [5-7]. Stimulation of the ADRB3, which are abundant in visceral adipose tissue [8], activates adenylatecyclase, which in turn increases the amount of intracellular cAMP and thus causes enhanced lipolysis in white adipose tissue and free fatty acids delivery to the portal vein [9]. Thus, ADRB3 gene expression plays an important role in lipolysis [10].

Theoretically the functional alterations in ADRB3 may promote the development of obesity and insulin resistance (IR) [11,12]. Missense mutation in the ADRB3 causes the substitution of the coding sequences from tryptophan into arginine in 64^th ^position, and this point mutation is able to influence receptor's affinity to norepinephrine and its interaction with Gs proteins in adipocytes [13]. Some studies had shown the associations between Arg64 variant of the ADRB3 gene and early development of type 2 DM and decreased early insulin response to glucose load [14], and, some studies with cellular transfection had shown an association with decreased glucose dependent insulin secretion [15].

Thereby ADRB3 gene is involved either directly or indirectly in lipid and glucose metabolism processes and may influence on endogenous energy balance and body mass regulation. At the same time, data about Trp64Arg polymorphism of the ADRB3 association with MS are inconsistent. Thus, approximately half of the studies found a positive association of the Trp64Arg polymorphism of the ADRB3 gene with obesity and IR [13,16-19], whereas the other studies didn't find any relationships between the presence of such polymorphism and obesity, type 2 DM or AH [20-22]. Such discordant results may be partially explained by ethnicity, age, or population differences in studied samples.

Previously Trp64Arg polymorphism of the ADRB3 gene had not been studied in the Kyrgyz population. The aim of this work was to study a possible association between this genetic polymorphism and MS components in an ethnic Kyrgyz group.

## Materials and methods

### Study population

Inclusion criteria: ethnic Kyrgyz people over 30 years of age who were permanent residents at the time, non consanguine to each other, and willing to participate in the study.

Exclusion Criteria: surgical intervention within a month prior to study enrollment, severe kidney and/or liver disease, thyroid dysfunction, alcohol abuse, treatment with insulin, hypolipidemic and corticosteroid medications, pregnancy and lactation.

National Center of Cardiology and Internal Medicine and Kyrgyz State Medical Academy Ethics Committee approved the study protocol. All participants were informed about the aim and study protocol and gave written consent prior to enrollment.

Finally, 213 volunteers (145 men and 68 women) aged 30-73 (mean age 50.7 ± 7.6) years were included in the study.

All subjects completed the medical examination. Blood pressure (BP) was measured using standard sphygmomanometer in the sitting position after a 10 minute rest. Anthropometric data included body mass index (BMI) as a weight (kg) to height (m^2^) ratio, waist circumference (WC) and hip circumference (HC) measurements. Modified Adult Treatment Panel III criteria were used for the MS diagnosis. [1].

### Laboratory tests

Blood collection from the cubital vein took place after 12 hours of fasting in the morning. Laboratory tests included fasting plasma glucose (FPG), lipid panel (total cholesterol (TC), triglycerides (TG), high density lipoprotein cholesterol (HDL-C)) and immunoreactive insulin levels. Low density lipoprotein cholesterol (LDL-C) was calculated by Friedwald W. formula [23]. IR index - HOMA was calculated as serum insulin(μIU/ml)x plasma glucose (mmol/l)/22.5. HOMA index ≥2.77 was considered as IR presence. Lipid panel analysis was performed on «Sinhron CX4-DELTA» biochemical autoanalysator («Beckman», USA).

### Genotyping

Genetic tests included DNA extraction with subsequent determination of Trp64Arg polymorphism of the ADRB3 gene. DNA was extracted from blood cells using «Nucleon BACC3» kit ("Amersham Pharmacia Biotech", Sweden). Trp64Arg of the ADRB3 polymorphism was detected using Restriction Fragment Length Polymorphism (RFLP) method. Polymerase chain reaction (PCR) was carried out on "Hybaid" amplificator (HBPX 220, Great Britain) with specific primers (F - CGCCCAATACCGCCAACAC and R - 3 CCACCAGGAGTCCCATCACC) and subsequent restriction of received PCR products by «BstOI» enzyme («Promega», USA). The received restriction fragments - Arg 64Arg 161 n.p., Trp64Arg 161+99+62 n.p., Trp64Trp np. were divided by electrophoresis in 3% Agar gel. Gel scanning and analysis of received results were made on image-densitometer «Fluor-S Multimager» ("Bio-Rad", USA).

### Statistical analysis

The "SPSS v. 17.0" and "Graph Pad PRIZM 5" software were used for statistical analyses. Student t test and Mann-Whitney U - test were used to assess differences between continuous variables with normal and nonparametric distribution respectively. The x^2 ^test was carried out to study an association between categorical variables. The data are presented as mean ± standard deviation (sd) for variables with normal distribution and median (25th and 75th percentile) for variables with nonparametric distribution. The logistic regression analysis was performed to test the potential independent association between Arg64 allele and obesity, AO, type 2 DM and AH. The p value < 0.05 was considered as a cut off for statistical significance.

## Results

ADRB3 genotypes were found to be in Hardy-Weinberg equilibrium. The frequency of Arg64 allele was 0.239 (0.269 and 0.176 in men and women respectively), ADRB3 genotype distribution forTrp64 homozygotes, Trp64Arg heterozygotes and Arg64 homozygotes were 54.5% (n = 116), 43.2% (n = 92) and 2.3% (n = 5) respectively. The Arg64Arg was rarely present in this sample, so all carriers of Arg64 allele were united in one group.

General characteristics of the included participants are present in Table 1.

**Table 1:** General characteristics of studied patients and correlation with risk factors:

Parameters	Group #1 Trp64Trp (n = 116)	Group # 2 Trp64Arg + Arg64Arg (n = 97)	P
Sex (Male)	59%	78%	0.004

Age, years	51.0 ± 7.9	50.4 ± 7.3	ns

Obesity, %	29	55.2	0.00009

BMI, kg/m^2^	27.7 ± 4.5	29.4 ± 4.9	0.008

AO, %	54.3	70.1	0.01

WC, cm	94.9 ± 13.2	101.7 ± 14	0.0004

WC/HC	0.95 ± 0.13	1.0 ± 0.14	0.028

AH, %	32.7	48.5	0.019

SBP, mmHg	134.3 ± 23.4	136.1 ± 24.1	ns

DBP, mmHg	84.8 ± 13.6	87.1 ± 13.2	ns

Type 2 DM, %	10.3%	24.7%	0.005

Fasting glycemia	5.9 ± 1.9	6.05 ± 2.02	ns

Smoking, %	24.7	25	ns

IR, %	21.3%(n = 80)	28.3% (n = 46)	ns

MS, %	37.9%	49.5%	ns

ns -non significant;

Both groups were matched for age and smoking status, but it is noteworthy that there were more males in the Group #2. Obesity (p < 0.00009) and AO (p < 0.01) were more prevalent in Trp64Arg and Arg64Arg genotypes, rather than in Trp64Trp genotype. Thus, BMI, WC and waist to hip ratio (WC/HC) were higher in the Group 2 (Table 1). Comparative associations of the ADRB3 genotypes with obesity parameters are present in the Figure 1.

**Figure 1:**
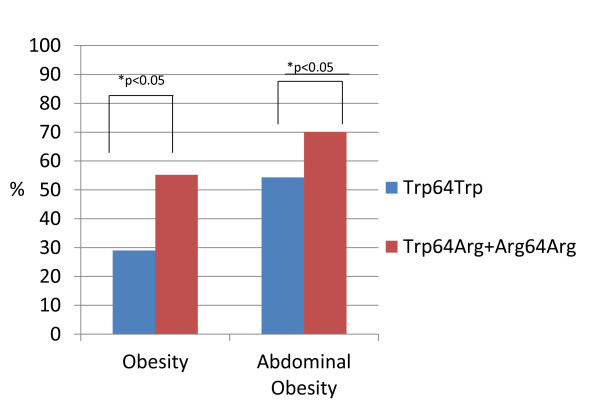
**ADRB3 genotypes and obesity parameters**.

AH and type 2 DM also were more frequent in the second group, but the differences in systolic and diastolic BP and fasting glucose plasma level between the two groups were not statistically significant. Individuals from Group #2 comparing to the Trp64Trp genotype carriers had a trend towards higher insulin levels and higher frequency of IR and MS, but aforementioned parameters did not reach statistical significance (table 2).

**Table 2:** Laboratory test results and relationship with MS and IR.

Parameters	Group #1 Trp64Trp (n = 116)	Group #2 Trp64Arg + Arg64Arg (n = 97)	P
TC, mmol/l	5.14 ± 1.08	4.92 ± 0.9	ns

LDL-C, mmol/l	3.23 ± 0.92	3.12 ± 0.8	ns

HDL-C, mmol/l	1.12 ± 0.34	1.02 ± 0.33	0.03

TG, mmol/l	1.36(0.92; 2.1)	1.42 (1.08; 2.22)	ns

Insulin, μIU/ml	6.65 (4.8; 9.89) (n = 80)	8.28(4.24; 11.8) (n = 46)	ns

ns -non significant

**Figure 2:**
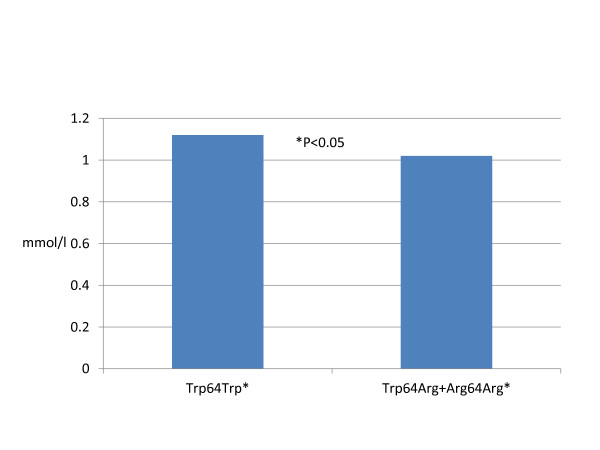
**ADRB3 and HDL-C level**.

Lipid profile components except the HDL-C level were approximately identical among both groups. HDL-C level was lower in Arg64 allele carriers (p < 0.03). Comparative associations of the ADRB3 genotypes with HDL-C level are present in the Figure 2.

The results of x^2 ^tests demonstrated the positive association between carrying the Arg64 allele and main MS components such as obesity (x^2 ^= 17.49; p < 0.0001), abdominal obesity (AO) (x^2 ^= 5.56; p < 0.018), AH (x^2 ^= 5.5; p < 0.019) and type 2 DM (x^2 ^= 7.8; p < 0.0053) (table 3).

**Table 3:** Trp64Trp, Trp64Arg and Arg64Arg genotypes of the B3AR gene and their correlation with AO, AH and DM type 2.

	Trp64Trp,n(%), n = 116	**Trp64Arg andArg64Arg**, n (%), n = 97	x^2^; p
Obesity:			
yes	34 (29.3)	56 (57.7)	x^2 ^= 17.49; p < 0.0001
no	82 (70.7)	41 (42.3)	

AO:			
yes	63 (54.3)	68(70.1)	x^2 ^= 5.56; p = 0.018
no	53 (45.7)	29 (29.9)	

AH:			
yes	39 (33.6)	48 (49.5)	x^2 ^= 5.5; p = 0.019
no	77 (66.4)	49 (50.5)	

DM 2:			
yes	12 (10.3)	24 (24.7)	x^2 ^= 7.8; p = 0.0053
no	104 (89.7)	73 (75.3)	

In a forward stepwise logistic regression model that included obesity as a dependent variable and the polymorphism of the ADRB3 (with Trp64 allele as reference) and sex as independent variables, only the presence of the Arg64 allele reached statistical significance (OR 3.16; 95% CI 1.79-5.56). In the same regression model with AO as the dependent variable the similar results were obtained (OR 1.97; 95% CI 1.12-3.48). For AH and type 2 DM after including only sex and polymorphism of the ADRB3 as independent variables the Arg64 allele increased the odds ratio for AH (OR 1.92; 95% CI 1.1-3.6) and for type 2 DM (OR 2.85; 95% CI 1.34-6.06), but after adjusting for obesity the role of Arg64 allele lost its statistical significance in both models.

## Discussion

In this study, we found that among ethnic Kyrgyz people the Trp64Trp genotype was more frequent and was discovered in 116 studied persons (54.5%). The Arg64Arg genotype was present only in 5 participants. The presence of Arg64 allele in our study showed a strong association with obesity and AO as well as greater WC and BMI, even after adjustment for gender.

It is well known that obesity is one of the most important risk factors for type 2 DM development, reflecting not only severity and duration of obesity, but also fat distribution [3]. From a theoretical point of view, it seems plausible that altered function of ADRB3 may promote the development of obesity through enhanced lipid accumulation in white adipose tissue and decreased biological energy expenditure from brown adipose tissue as a result of decreased lipolysis [18,19]. It was shown that ADRB3 mRNA is abundantly present in visceral adipose tissue, compared with subcutaneous fat [9]. Diminished lipid oxidation may serve as a predictor of subsequent increase of fat amount in the human organism [24]. Some studies had shown associations between obesity and Trp64Arg polymorphism of the ADRB3 gene [25], but not others [26,27]. In studies focused on ADRB3 gene polymorphism and BMI, there was a stronger association between in Asian population, compared to Caucasians [28,29]. The Chinese researchers had shown that ADRB3 R64 allele was associated with increased BMI and weight [30] and research group from Poland had found the protective effects of the ADRB3 Arg64 polymorphism against metabolic disorders [31].

Results of the studies focused on Trp64Arg carrier state association with MS are also controversial. Some found a correlation between this polymorphism and body weight and BMI variables [32], whereas others detected that W64 allele homozygotes of the ADRB3 are relatively protected against metabolic abnormalities [30]. In the Quebec Family Study researchers did not find any differences in glucose, insulin levels and BP values in studied groups with and without Arg64 allele [22]. Japanese researchers found ADRB3 polymorphism association with IR, but not with dyslipidemia [33]. Also prior investigations did not find statistically significant relationships between ADRB3 genotype and type 2 DM [13,17,34]. Nevertheless it reached statistical significance in the meta analysis performed by Fujisawa T et al. [33]. In our study the frequency of type 2 DM in Trp64Arg and Arg64Arg group was higher but the association lost its significance after adjustment for obesity.

Experimental data clearly shows that, besides its effect on lipolysis and biological energy production, ADRB3 may modulate peripheral vascular tone and increase the BP [35]. Some clinical studies pointed on possible relationship between AH and Trp64Arg polymorphism [17,35,36], as well as relationship between this genotype and higher mortality among hypertensives [37]. In our study, we found an association of Trp64Arg carrier state with AH (p < 0.019). After logistic regression analysis, including gender and ADRB3, the association was still statistically significant, but after including obesity in the list, ADRB3 lost its significance. It seems that AH as well as most other disorders are polygenic in nature where many genes and environmental factors play a role in the pathogenesis. It may be speculated that Arg64 allele may indirectly affect AH risk through the development of obesity. In a recently published Japanese work it was clearly shown that Trp64Arg polymorphism is associated with cardiovascular risk among hypertensive [31]. Besides the ADRB3, some genetic variants of the cardiac B 1 adrenoreceptor may play a role in the development of left ventricular hypertrophy in patients after acute coronary event in patients without DM or AH [38]. This highlights the possibility of altered sympathetic signaling in the pathogenesis of cardiovascular disease.

Data on the ADRB3 gene mutation effect on lipid metabolism are inconsistent and heterogeneous. Some studies did not find any correlation between Arg64 allele and serum lipids [17,22]. On the other hand, a study conducted on a young Danish population showed, that this mutation had an associations with hypertriglyceridemia and increased LDL-C levels [39]. Recently published data in Spain about Trp64Arg carrier state in hypertensive cohort had shown associations with higher BMI and TC level [40]. In our sample, Trp64Arg carriers had lower HDL-C levels, but other cholesterol metabolism parameters did not reach statistical significance.

## Conclusion

Our work was the first in studying Trp64Arg polymorphism among ethnic Kyrgyz people, where we found an association of this ADRB3 gene polymorphism with components of MS, such as obesity, AO, and decreased HDL-C level. This study and its findings may be useful and provide some basic information regarding our understanding of complex pathological interconnections between metabolic heritage and modern environment.

## Abbreviations

**ADRB3: **B3 adrenoreceptor; **AH: **arterial hypertension; **AO: **abdominal obesity; **BMI: **body mass index; **DBP: **diastolic blood pressure; **DM: **diabetes mellitus; **HC: **hip circumference; **HDL: C-**high density cholesterol; **IR: **insulin resistance; **LDL-C: **low density cholesterol; **MS:**metabolic syndrome; **SBP: **systolic blood pressure; **TC: **total cholesterol; **TG:**triglycerides; **WC: **waist circumference

## Competing interests

The authors declare that they have no competing interests.

## Authors' contributions

All authors contributed equally during the investigation process and article writing. All authors participated in manuscript discussion. All authors have read and approved the final manuscript. AEM performed English translation and revision of the manuscript.
